# Prevalence and risk factors associated with hypertension among adults in a rural setting: the case of Ombe, Cameroon

**DOI:** 10.11604/pamj.2019.34.147.17518

**Published:** 2019-11-14

**Authors:** Fuh Princewel, Samuel Nambile Cumber, Judith Anchang Kimbi, Claude Ngwayu Nkfusai, Elsie Indah Keka, Vecheusi Zennobia Viyoff, Terence Epie Beteck, Fala Bede, Joyce Mahlako Tsoka-Gwegweni, Eric Achidi Akum

**Affiliations:** 1 Department of Microbiology and Parasitology, Faculty of Science, University of Buea, Buea, Cameroon; 2 Faculty of Health Sciences, University of the Free State, Bloemfontein, South Africa; 3 Section for Epidemiology and Social Medicine, Department of Public Health, Institute of Medicine (EPSO), The Sahlgrenska Academy at University of Gothenburg, Box 414, SE – 405 Gothenburg, Sweden; 4 School of Health Systems and Public Health, Faculty of Health Sciences, University of Pretoria Private Bag X323, Gezina, Pretoria, South Africa; 5 HIV Free Project, Cameroon Baptist Convention Health Services, Yaounde, Cameroon; 6 Centre for Tropical Medicine and Global Health, Nuffield Department of Medicine, University of Oxford, Oxford, United Kingdom

**Keywords:** Hypertension, high blood pressure, prevalence, risk factors, Ombe, rural community, Cameroon

## Abstract

**Introduction:**

High blood pressure is the most common cardiovascular disorder affecting approximately one billion people globally and remains a major contributor to the global burden of non-communicable diseases and mortality. Hypertension, once rare in traditional African societies, is now a major public health problem probably because of a rise in its risk factors. In sub-Saharan Africa, an estimated 74.7 million individuals live with hypertension. This study was designed to determine the prevalence and identify risk factors associated with hypertension in adults aged 21 years and above in Ombe village, a rural Cameroonian setting in sub-Saharan Africa.

**Methods:**

This study was a cross-sectional community based survey from March to September 2016 (seven months) in the village of Ombe, a rural community in the southwest region of Cameroon. Following ethical clearance from the Institutional Review Board (IRB) of the Faculty of Health Science, University of Buea and administrative authorization, 243 participants (141 males and 102 females) through multi-stage sampling were randomly selected to take part in the study following consent which was voluntary and without any form of coercion. The principal research instrument was a questionnaire adapted from the WHO STEPwise approach to chronic disease risk factor surveillance- Instrument v2.1 which was administered to participants. The self-administered questionnaire collected socio-demographic information, data related to knowledge and practices related to hypertension, anthropometric data (weight and height from which the body mass index (BMI) was calculated). The blood pressure of study participants was measured. Data was entered using Microsoft Excel, then imported and analysed in SPSS v22.0. Frequencies and percentages were determined for categorical variables. Means and standard deviations (mean ± SD). Univariate and multivariate logistic regression analysis were used to investigate factors associated with high blood pressure (hypertension).

**Results:**

The results showed that 19.8% of the adult population had hypertension. Of the 243 respondents, 77.7% understood what is hypertension, 85% indicated that they could tell some consequences of high blood pressure (they indicated hypertension affects the heart, brain and kidneys) and 63.3% of study participants had never checked their blood pressure. Age greater than 40 years, harmful alcohol intake for more than 10 years, physical inactivity and obesity (BMI ≥ 25Kg/m^2^) were variables associated with hypertension on univariate analysis. Following multivariate analysis, independent risk factors for hypertension in our study were: physical inactivity (Adj. OR 2.6, 95%CI: 1.3-4.4, p = 0.021), regular alcohol consumption for more than 10 years (Adj. OR 2.9, 95%CI: 1.6-5.1, p = 0.014) and being older than 40 years of age: Adj. OR 2.5, 95%CI: 1.02-4.1, p = 0.002 in age category 41-60 years and this age related risk was even higher in persons older than 60 years of age Adj. OR 4.5, 95%CI: 2.1-6.3, p = 0.002.

**Conclusion:**

The findings of this study showed the prevalence of high blood pressure among adults in Ombe (a rural community in Cameroon) was 19.8%. Old age, alcohol consumption, and physical inactivity were independent risk factors for hypertension. Despite the population demonstrating knowledge about hypertension and its possible poor consequences on health, less than half had ever gone for blood pressure checks. Interventions to improve physical activity, reduce alcohol consumption and boost health seeking (high blood pressure screening) behaviour will be beneficial as preventive measures in combatting hypertension.

## Introduction

High Blood Pressure (HBP) or hypertension (HTN) is a chronic medical condition in which the blood pressure is elevated [1]. High blood pressure is the most common cardiovascular disorder affecting approximately one billion people globally and remains the leading single contributor to global burden of disease and mortality [2]. In 2000, there were an estimated 972 million people with HTN, 65% of whom lived in the developing world with the number predicted to grow to 1.5 billion by 2025. In Africa, however, more than 40% (and up to 50%) of adults in many countries are estimated to have high blood pressure [3]. In sub-Saharan Africa, an estimated 74.7 million individuals are hypertensive [3]. In most African countries and Cameroon in particular, HTN is the most common non-communicable disease [4]. In Cameroon, surveys on hypertension report a prevalence varying from 12 to 22% in those above 25 years [5]. A study conducted in Douala Cameroon to check the burden of HTN and related risk factors in urban sub-Saharan Africa, revealed a prevalence of 20.5% [6]. Hypertension is classified into essential (primary) or secondary hypertension based on the etiology. Primary or essential hypertension is the most common type, affecting 90-95% of hypertensive patients. It is defined as a rise of blood pressure of unknown origin (idiopathic). Secondary HTN is the increase in blood pressure caused by diseases. In addition, HBP can be regarded as mild or moderate. Several factors put people at risk of developing HTN and can be either modifiable or non-modifiable. Gender, age, race, and heredity are risk factors that cannot be modified. Modifiable risk factors include lifestyle related factors such as obesity, diet, physical inactivity, stress, the use of certain medications, smoking, excessive alcohol consumption. They could also be pathologic such as diabetes mellitus and dyslipidemias. The ultimate goal in the treatment of hypertension is to reduce the risk of cardiovascular event for individual patients and in the population as a whole. If left untreated, complications such as atherosclerosis, heart attack, stroke, enlarged heart or kidney damage may occur due to high blood pressure.

## Methods

### Study design

The study was a cross sectional community based study. It was carried out to determine the prevalence of high blood pressure (hypertension) and identify risk factors associated with the development of hypertension in an adult population (21 years and above) in Ombe. The study was conducted from March to September 2016.

### Setting

The study was conducted in Ombe, a rural settlement with a heterogeneous population and located in the southwest region of Cameroon. It is located at latitude 4° 00' 45" N and longitude 9° 19' 16" E. Ombe is made up of five quarters. The main occupation of residents in this area is farming. Ombe has a total population of about 500 inhabitants. Ombe has two seasons (rainy and dry) with varied topography made of hills and valleys through which the population moved to their various destinations on daily basis.

### Study population

The population of interest of the study were all adults residing in Ombe.

**Inclusion criteria:** adult of age 21 years and above; accepted (voluntarily) to take part in the study by signing a consent form; must have been residing in Ombe for at least two years in order to adapt to the way of life of Ombe village; be in good health.

**Exclusion criteria:** pregnant and breastfeeding women were excluded; critically sick patients were excluded; visitors and temporary residents in Ombe were not included in this study.

### Sample size determination and sampling method

Sample size was determined based on the Taro Yamane’s approach [7].

n = N / 1+N (e) ^2^

Where, n = the expected sample size, N = finite population out of which the sample size is drawn and e = level of precision. The target population for this study is 600 [8] and precision of 5% (e = 0.05)

n = 600 /1+600(0.05)^2^ = 243 participants

A multi-stage sampling was used to randomly select 243 study participants. In the first step, 5 households were randomly selected from each of the 5 quarters that constituted the village of Ombe. Within each household, two adults (21 years and older) meeting inclusion and exclusion criteria were randomly selected. Where there were just two, they were all included and where there was just one another household was randomly selected from another was sampled.

### Study variables and data collection procedure

**Study procedure:** data for this study was collected using a questionnaire adapted from the “WHO STEP wise approach to chronic Disease Risk Factors Surveillance- Instrument v2.1” which was administered to participants to permit study objectives to be met. The questionnaire collected data on socio-demographic information, knowledge and practices about hypertension, lifestyle, blood pressure measurements and anthropometric data (weight, height, body mass index, BMI). It was pretested in another rural community (Tombel). The questionnaire was administered to study participants by a researcher and anthropometric parameters were measured by the same researcher. The researcher was trained on how to take anthropometric measurements and how to recalibrate measuring instruments after use.

### Study variables

**Blood pressure:** the outcome variable for our study was hypertension. Hypertension (HTN) for the purpose of our study was defined as that level of arterial blood pressure where systolic level was = 140mmHg and/or diastolic level = 90 mmHg [3]. The blood pressure of the study participants were measured using a manual blood pressure machine. The systolic and diastolic pressure were measured using a manual aneroid sphygmomanometer (OMRON, HEM 712C). Participants were in an upright sitting or reclining positions and the recordings were taken after a period of 20 minutes’ rest. Four recordings were taken two in each arm at an interval of 5 minutes each and their mean value for both arms calculated. Systolic BP was measured at the appearance of the Korotkov's sounds (Phase I) and diastolic BP was taken at the point of disappearance of the sounds (Phase V).

**Height:** the height (m) of the study participants were measured using a calibrated meter rule to the nearest 0.1 centimeters (cm). Participants removed their shoes prior to height measurements and measurement was done from heel to head crown with the participant standing straight upright.

**Weight:** the weight (kg) of the volunteer study participants was measured using a scale to the nearest one kilogram. Weight was measured without shoes and with minimum clothing.

**Body Mass Index:** this was calculated from the body weight and height using the calculations:

$$\text{BMI=}\frac{Weight(Kg)}{Height(m)}$$

The BMI was categorized into Obese (BMI = 25Kg/m^2^) or Non-obese (BMI < 25Kg/m^2^). Salt intake can be measured either by 24 hour dietary recall method or weighment method or by simple food frequency.

### Ethical considerations

Administrative authorizations were obtained from the Regional Delegation of Public Health for the southwest Region, the District Medical Officer of the Tiko Health District and the local administration. Ethical clearance was sought and obtained from the Institutional Review Board, University of Buea (Ref: 2016/089/UB/SG/IRB/FHS). This was a non-invasive study which entailed no risks or harm to the participants but required them to reveal some personal information. Consent was sought from participants following detailed explanation of the study with each person given an opportunity to have any doubts clarified. This was a voluntary process without any form of coercion. Participants were free to withdraw from the study and any point in time and have their information collected not used. Any person identified with high blood pressure was counseled and referred to get appropriate care if he/she was not already receiving one. Confidentiality, anonymity and privacy of all information were guaranteed at all the levels of this study. Patients diagnosed with hypertension were referred to hospital for long term management.

## Results

### Socio-demographic characteristics of the study participants

A total of 243 participants took part in the study. Of these, the age group 21-40 years was the most frequent (67.1%). Males constituted 58% of study participants, majority were farmers (63.5%) with 59.6% in the low socio-economic status (SES) category. Christians made up 92.5% of participants.

### Attitude and practice of community respondents about hypertension in Ombe

The prevalence of hypertension was 19.8%. Of all the 243 study participants who took part in the study, 63.4% consume alcohol while 10.3% were smokers and 135 carried out moderate physical activities (Table 1). Of all the study participants, 208 consume fruits and vegetable and 181 stress up themselves. Out of the 243 study participants, 67.5 % have heard of hypertension, 63.8% have never done any test for hypertension and 12.8 % have been told that they are hypertensive.

**Table 1:** Socio-demographic characteristics of 243 study participants recruited from the community, Ombe, 2018

Characteristic	Groups	Frequency	Percentage (%)
**Age (years)**	21 – 40	163	67.1
	41 – 60	60	24.7
	60+	20	8.2
**Marital status**	Single	113	46.9
	Married	101	41.9
	Widow (er)	29	11.2
**Gender**	Male	141	58.0
	Female	102	42.0
**Level of Education**	Primary	74	30.6
	Secondary	108	44.7
	Tertiary	61	24.7
**Occupation**	Farmer	154	63.5
	Employed	47	19.1
	Students	42	17.4
**Socio-economic status (SES**)	Low SES (< 50,000) frs	145	59.6
	Middle SES (50,000-100,000) frs	57	23.5
	High SES (>100,000) frs	41	16.9
**Religion**	Christians	223	92.5
	Muslims	11	4.1
	Others	9	3.3

### Risk factors associated with hypertension

The logistic regression analysis showed that age above 40 years was associated with an increased risk of hypertension (Adj. OR 2.5, 95%CI: 1.02-4.1, p = 0.002) relative to persons less than 40years of age. This risk increased with persons older than 60years of age (Adj. OR 4.5, 95%CI: 2.1-6.3, p = 0.002) (Table 2). Concerning biological and behavioral determinants of hypertension (Table 3), regular alcohol consumption for more than 10 years doubles the risk of developing hypertension (Adj. OR 2.9, 95%CI: 1.6-5.1, p = 0.014) Obesity defined as a BMI = 25Kg/m^2^ was associated with an increased risk of developing hypertension compared to non-obese participants (OR 1., p = 0.01). This effect however, was not significant in the multivariate logistic regression analysis (Adj. OR=1.3, 95% CI: 0.74-2.01 and p = 0.089). Likewise, physical inactivity was a risk factor for hypertension in our study (Adj. OR 2.6, 95%CI: 1.3-4.4, p = 0.0021) (Table 2).

**Table 2:** Socio-economic characteristics of study participants from Ombe Fako Division

Characteristic	Group	Frequency	Percentage (%)
Physical activities	Little	8	3.3
	Moderate	135	55.6
	Much	100	41.1
Alcohol Intake	Yes	154	63.4
	No	89	36.6
Smoking	Yes	25	10.3
	No	218	89.7
Salt Intake	Little	03	1.2
	Moderate	148	60.9
	Much	85	35.0
	Too Much	07	2.9
Consumption of Fruits and Vegetables	Yes	208	85.6
	No	35	14.4
Do you Stress up yourself	Yes	181	74.5
	No	62	25.5
Have you ever heard of Hypertension	Yes	164	67.5
	No	79	32.4
Have you ever test for HTN	Yes	88	36.2
	No	155	63.8
Have you ever been told you have HTN	Yes	31	12.8
	No	212	87.2

**Table 3:** Distribution of different category of blood pressure by socio demographic factors of study participants in Ombe

Variables	OI (%)	Univariate logistic regression			Multi-variate logistic regression		
		OR	95% CI	p-value	Adj. OR	95% CI	p-value
Age*							
21-40 years (n=163)	67.1%	1		**0.001**	1		**0.020**
41-60 years (n= 60)	24.7%	2.30	1.21-4.01		2.50	1.02-4.10	
>60 years (n=20)	8.2%	4.40	2.44-5.71		4.50	2.50-6.30	
Regular alcohol consumption for > 10 years *							
No (n=89)	36.6%	1		**< 0.001**	1		**0.014**
Yes (n=154)	63.4%	2.80	1.74-4.90		2.90	1.60-5.10	
**Do you Stress most of the time?**				**0.780**			**0.723**
No (n=62)	25.5%	1					
Yes (n=181)	74.5%	1.50	0.74-1.65		1.10	0.63-1.91	
Does your work involve vigorous physical activity*				**<0.001**			**0.021**
Yes (n=100)	41.1%	1			1		
No (n=143)	58.8%	2.30	1.25-4.51		2.60	1.30-4.40	
Body Mass Index (BMI)*		0.010					0.089
BMI < 25Kg/m^2^ (n=196)	80.7%	1			1		
BMI ≥ 25Kg/m2 (n=47)	19.3%	1.70	1.15-3.10		1.30	0.74-2.01	

### Knowledge of participants on hypertension

As far as the Knowledge, Attitude and Practice of hypertension were concerned, of the 243 respondents, 188 (77.7%) were familiar with hypertension and knew what hypertension was all about. One hundred and fifty-two (63.3%) participants had never had their BP checked whereas 89 (36.7%) had had their blood pressure checked at least once. On the knowledge on how often do you think people of your age group should check their BP, 161 (66.8%) indicated that it should be regular at least once a year. Reasons advanced for not regularly going for BP check at least annually were: 99 (41.3%) did not think it was important, 30.5% mentioned time constrain and 28.2% said they did not know where to get such services.

### Attitude and practice of participants on hypertension

Amongst all the respondents, 215 (89.5% mean percentage) participants say high blood pressure is a health problem and likely to cause other health problems. Above 85% of all participants indicated that hypertension affects the heart, brain and kidneys. Poor dietary practices (excess salt consumption) was identified by 208 (86.5%) of participants to be a cause for high blood pressure. A good number of participants, 192 (81.6%), correctly answered high blood pressure as being treatable as they said medications effectively lower blood pressure (Table 4).

**Table 4:** Knowledge attitude and practice of hypertension

Characteristic	Group	Number	Percentage(95% CI)
How much do you know about high blood pressure (243)	Nothing at all	54	22.3 (26.4 – 38.5)
	Familiar with it	188	77.7 (61.2 – 73.4)
Have you ever had your BP checked (243)	No	154	63.3 (56.2 – 69.5)
	Yes	89	36.7 (30.2 – 43.5)
How often do you think people of your age group should have BP check	Only when they are sick	80	33.2 (30.5 – 38.2)
	Regularly (once a year)	161	66.8 (60.2 – 71.5)
Main reason for not checking once blood pressure (N=240)	Don’t know where to access the service	68	28.2 (25.3 – 32.3)
	Don’t think it is important	99	41.3 (38.0 – 46.3)
Does HBP cause other health problems	No	19	8.0 (6.2 – 10.5)
	Yes	215	89.5 (80.2 - 94.5)
	don’t know	6	2.5 (1.02 – 4.1)
Does HBP affects the heart	Yes	222	91.2 (90.1 – 94.3)
	Don’t know	4	1.7 (1.2 – 3.2)
	No	17	3.0 (1.5 – 5.2)
Does HBP affects the brain	Yes	228	90.3 (87.1-94.2)
	No	7	3.0 (1.5-5.2)
	Don’t know	15	6.7 (4.0 – 8.3)
**Characteristic**	**Group**	**Number**	**Percentage(95% CI)**
Does HBP affect the kidney	No	12	5.0 (2.8 – 7.5)
	Yes	208	86.5 (82.0 – 90.2)
	Don’t know	20	8.5 (6.7 – 11.5)
	No	11	4.5 (2.1 – 6.3)
Can eating food with a lot of salt affect blood pressure	Yes	208	86.5 (82.0 – 91.2)
	Don’t know	22	9.0 (7.2 – 11.5)
	Not effective	28	12.0 (10.4 – 14.3)
The effectiveness of medication in reducing BP? (N=235)	Effective	192	81.5 (79.0 – 85.1)
	Don’t know	15	6.5 (4.3 – 8.3)
The effectiveness of exercise in reducing BP?(N=240)	Not effective	20	8.2 (6.5 – 10.3)
	Effective	210	87.6 (84.2 – 88.2)
	Don’t know	10	4.2 (2.3 – 6.2)

## Discussion

In this study of 243 adults of age 21 years and above, the prevalence of hypertension was 19.75%. This result has an isolated systolic, isolated diastolic and mixed hypertension of 20.8%, 22.1% and 56.3% respectively. The prevalence of hypertension was found to be higher in males than in females. This is consistent with the reported prevalence of hypertension in rural areas of India, Bangladesh and Pakistan, which is in the range of 4.5 - 22% [8-10] and 14.6% in Cameroon [10]. The age group of 60+ years had the highest rate of hypertension. This could be because this age group was mostly constituted of farmers. A similar observation to the findings of this study has been reported. Hypertension was more prevalent among older people. This is in line with [6], who said, the prevalent rate of hypertension increases with increase in age in all adults.

Though the prevalence of hypertension was not significantly different in both males and females, mean systolic and diastolic blood pressures were significantly higher in males than in females and the frequency of participants with systolic blood pressure (SBP) and diastolic blood pressure (DBP) were higher in males than in females. A higher proportion of normotensive participants were males. This is consistent with a study carried out in rural India [11, 12]. Previously, gender differences in blood pressure have been attributed to the differences in dietary habits, lifestyle choices, physical activity level and some genetic polymerisms [12]. This study reveals a high level of physical activities in women than in men. Despite the high level of physical activities in female than males, more females were obese than males as per this study. The higher prevalence of obesity in females than males is coherent with studies carried out by [4, 10, 13].

Females were more physically active than males probably because more females than males were involved in farming that was equated to moderate activity. A significant proportion of hypertensive participants were sedentary. This is similar to a study in Cameroon carried out by [13] which revealed that the incidence of hypertension increased in individuals with sedentary lifestyle. Systolic and Diastolic blood pressure was observed to increase with BMI; hypertensive participants had a higher BMI than non-hypertensive participants. This indicated a positive association between hypertension and BMI. Other studies had shown similar results [4, 14]. HTN among smokers and individuals, who ate less than 5 servings of fruits and vegetable, was less significant. BP was not associated with salt intake levels but was strongly associated with fasting blood sugar (FBS) levels and BMI. Although SBP and DBP did not increase significantly with increase in fasting blood glucose (FBG) concentration, hypertensive participants had a significantly higher mean fasting blood glucose compared to non-hypertensive participants. Thus, we could effectively deduce that fasting blood glucose correlated positively with hypertensive status.

There was no association between smoking and the development of high blood pressure. This results obtained differ from those obtained by [15, 16] who showed that the incidence of hypertension increased with the number of sticks of cigarette smoked per day. This may be due to a low population of smokers in our study population. This study revealed that regular alcohol consumption for more than ten years tripled the risk of developing hypertension. This positive association was coherent with studies by some authors, who showed that blood pressure was significantly increased by alcohol consumption. Alcohol was noticed to be the second most important risk factor for the development of hypertension in the Russian population [17]. Globally, the effects of regular alcohol consumption on the development of hypertension had been extensively studied [18, 19].

Knowledge on the risk factors of hypertension was above average, as most participants knew about hypertension and some had their blood pressure checked at least once. A majority of the participants were aware that blood pressure would cause other health problems like affecting the brain, heart and kidneys. Among the 243 participants, hypertensive participants were the more knowledgeable group. A possible reason why the hypertensive participants were the more knowledgeable group might be due to the education they received in the hospital concerning their health status when they enrolled for treatment. The other proportion with low level of awareness of hypertension and its risk factors in this community was similar to results obtained [20] in a study done in Yaounde, in which he concluded that there was a low level of awareness in the notion of hypertension within the population.

## Conclusion

This study showed that; the prevalence of hypertension in adults was 19.75% in this rural Cameroonian setting. Some risk factors associated with the development of HTN in adults are: age, regular alcohol consumption, level of physical activity, obesity, stress and fasting blood glucose concentration in blood. More than 60% of the study participants had knowledge on hypertension, practice save eating habits and exercise. Sensitization of the population on hypertension and its complications is important.

### What is known about this topic

- Hypertension as a causative factor of cardiovascular diseases;
- The risk factors and control of hypertension in developed countries as well as the attitude and practice of participants towards hypertension;
- Prevalence studies on hypertension in urban areas like the Centre and Littoral regions of Cameroon.

### What this study adds

- This study provides information about the prevalence and risk factors of hypertension in a rural community of Ombe;
- The study adds more knowledge on the attitude and practice of community respondents towards hypertension since little has been done in Cameroon;
- The need to reinforce sensitization, health information, mobilization and advocacy, which will be quite instrumental in creating awareness on the various ways of preventing and treating the disease in the community; constant epidemiological surveys by researchers are needed to monitor the disease.

## Competing interests

The authors declare no competing interests.
